# P-890. Exit Strategy: A Retrospective Analysis of Parenteral Antibiotic Stewardship at Discharge

**DOI:** 10.1093/ofid/ofaf695.1098

**Published:** 2026-01-11

**Authors:** Karan Raja, Rebecca Lee, Soo Kang, Mitesh Patel, Mona Philips

**Affiliations:** Clara Maass Medical Center, Belleville, NJ; Clara Maass Medical Center, Belleville, NJ; Clara Maass Medical Center, Belleville, NJ; Clara Maass Medical Center, RWJBarnabas Health, Belleville, NJ; Clara Maass Medical Center, RWJBarnabas Health, Belleville, NJ

## Abstract

**Background:**

Inpatient antibiotic stewardship programs (ASP) are associated with improved infection cure rates, as well as reduced risk of adverse drug events and antibiotic resistance development. However, there is often limited ASP presence at the discharge setting. Data has shown over 70% of parenteral antibiotic prescriptions at hospital discharge are excessively broad spectrum or have prolonged durations. Additionally, these patients’ discharge planning process is complex, prolonging length of stay (LOS). The objective of this gap analysis is to evaluate the inpatient LOS and antibiotic appropriateness of parenteral antibiotic therapy prescribed at discharge from an urban, non-teaching, community medical center.

**Methods:**

This IRB-approved retrospective cohort study evaluated adult patients discharged on parenteral antibiotics between January and March 2024. The primary outcome is LOS in days from hospital admission to discharge. We also evaluated antibiotic appropriateness, antibiotic days of therapy, vascular access devices, 30-day readmission rates, and discharge disposition. Antibiotic appropriateness was assessed using the validated National Antibiotic Prescribing Survey (NAPS) which stratifies regimens as appropriate (optimal or adequate), inappropriate (suboptimal or inadequate), or not assessable based on local treatment guidelines, literature references, and patient-specific factors. Inappropriate antibiotic regimens were further evaluated to identify specific opportunities for ASP intervention. Data were evaluated using descriptive statistics, including measures of central tendency and dispersion.

**Results:**

A total of 168 patients met inclusion criteria. The average LOS was 10.7±7.7 days. The proportion of appropriate and inappropriate antibiotic regimens were 52.8% and 38.8%, respectively. The most frequently identified opportunities for intervention included improving antibiotic selection, duration, and transition to oral antibiotics.

**Conclusion:**

This gap analysis illustrates discharge parenteral antibiotic stewardship metrics, including associated inpatient LOS and antibiotic appropriateness. Based on these findings, we aim to integrate ASPs in discharge planning to close these gaps and improve antibiotic utilization.

**Disclosures:**

All Authors: No reported disclosures

